# Supplementary material for: Reconstitution of human PDAC using primary cells reveals oncogenic transcriptomic features at tumor onset
Source: Nat Commun. 2024 Jan 27;15:818. doi: 10.1038/s41467-024-45097-2 (PMC10821902; doi:10.1038/s41467-024-45097-2)
Supplement: Supplementary file 1 — Supplementary Information [file 41467_2024_45097_MOESM1_ESM.pdf]

Reconstitution of human PDAC using primary cells reveals oncogenic transcriptomic features at tumor onset

Yi Xu[1#], Michael H. Nipper[1#], Angel A. Dominguez[1], Zhengqing Ye[2,3], Naoki Akanuma[1], Kevin Lopez[1], Janice J Deng[1], Destiny Arenas[1], Ava Sanchez[1], Francis E. Sharkey[4], Colin M. Court[5], Aatur D. Singhi[6], Huamin Wang[7], Martin E Fernandez-Zapico[8], Luzhe Sun[1], Siyuan Zheng[2,3], Yidong Chen[2,3], Jun Liu[1*] and Pei Wang[1*]

[1]Department of Cell Systems & Anatomy, [2]Greehey Children's Cancer Research Institute, [3]Department of Population Health Sciences, [4]Department of Pathology and Laboratory Medicine, [5]Division of Surgical Oncology and Endocrine Surgery, University of Texas Health Science Center at San Antonio, San Antonio, TX, 78229, USA.

[6]Department of Pathology, University of Pittsburgh Medical Center, Pittsburgh, PA 15213, USA.

[7]Department of Pathology, University of Texas MD Anderson Cancer Center, Houston, TX 77030

[8]Schulze Center for Novel Therapeutics, Division of Oncology Research, Mayo Clinic, Rochester, MN 55905, USA.

# These authors contribute equally

* Correspondence authors: Drs. Pei Wang and Jun Liu, Department of Cell Systems & Anatomy, University of Texas Health Science Center at San Antonio, 7703 Floyd Curl

Drive, San Antonio, TX, 78229-3900, USA. Phone: (210) 567-6796, E-mail: wangp3@uthscsa.edu, Liuj8@uthscsa.edu.

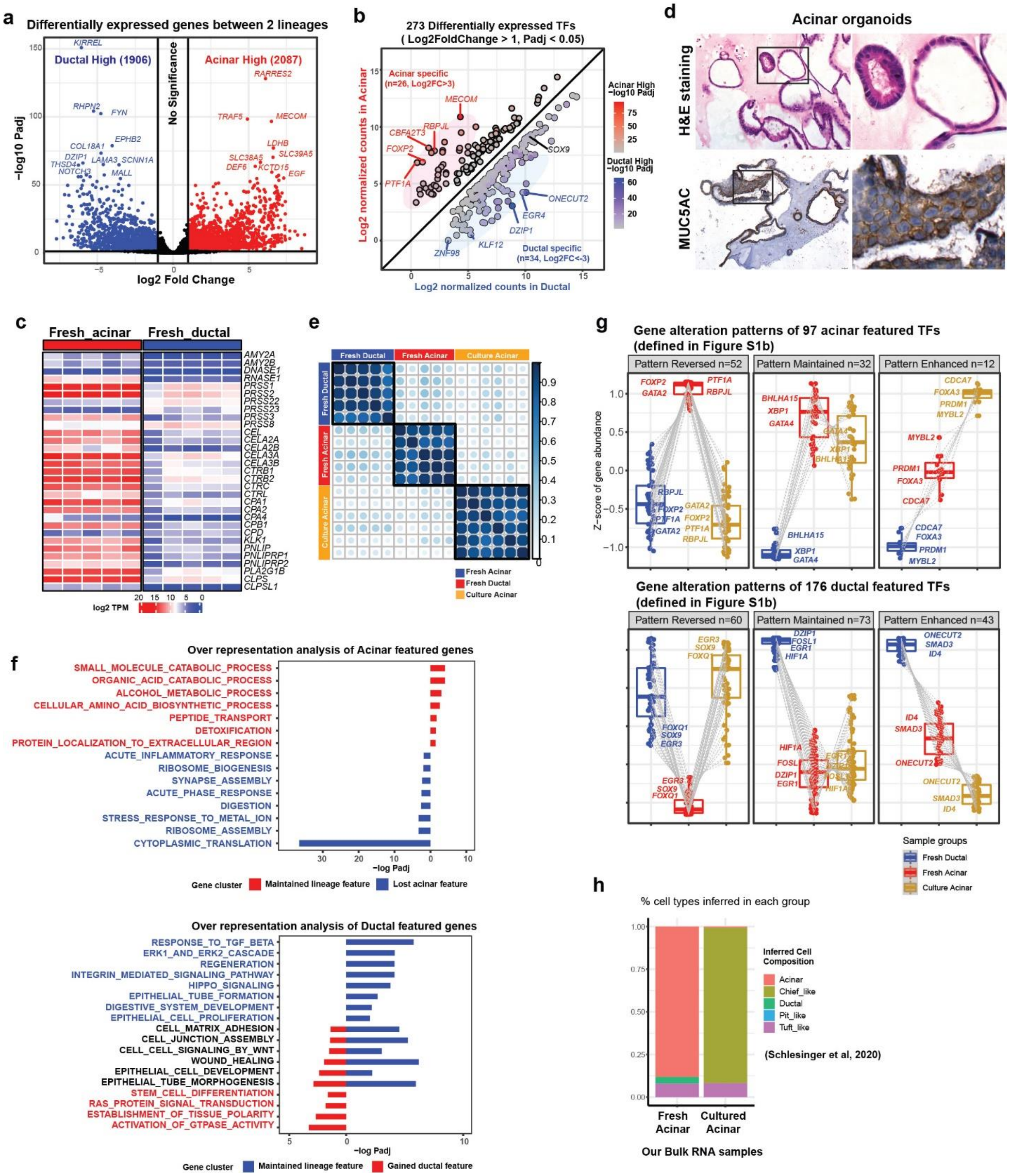

Figure S1. Molecular profiling of fresh and cultured human primary pancreatic exocrine cells. a. Volcano plot of the differentially expressed genes in fresh acinar (n=5) vs fresh ductal cells (n=5, fold change > 2, adj.p < 0.05, by negative binomial

Wald test using DESeq2 package in R software). **b.** Mean normalized expression of 273 differentially expressed transcription factors in sorted fresh acinar and ductal cells (fold change > 2, adj.p < 0.05). The shaded area includes the most significantly differentially expressed TFs in each lineage (log2 fold change > 3). **c.** Expression heatmap of genes encoding known pancreatic digestive enzymes in sorted fresh cells. **d.** H&E staining and MUC5AC staining in cultured acinar organoids. Each staining was performed in sections from 3 independent donor tissues with 8 fields of views for each. Scale bar: 50 µm. **e.** Pearson correlation plot of gene expression in fresh acinar cells (n=5), fresh ductal cells (n=5), and cultured acinar organoids (n=6). **f.** Gene ontology overrepresentation analysis of the two gene subgroups within the acinar signature gene cluster and ductal signature gene cluster described in Figure 1d. The analysis was performed using the clusterProfiler 4.4.4 package in R software with default settings. A significant enrichment was considered with multiple-test adjusted p-value < 0.05. **g.** Expression patterns of 97 acinar specific and 176 ductal specific transcription factors in fresh acinar, fresh ductal and cultured acinar cells. Each dot represents the mean expression of a transcription factor in the indicated group of samples. The middle line of box represents the median value of the expression of a group of transcription factors, the bounds of box represent the IQR, and the whiskers extend to 1.5 × IQR. **h.** Bar plot of inferred cell type composition in our fresh (n=5) and cultured acinar (n=6) samples using a previously characterized scRNA seq dataset as a reference. The bulk RNA sample deconvolution was performed by using a Multi-Subject Single Cell deconvolution method.

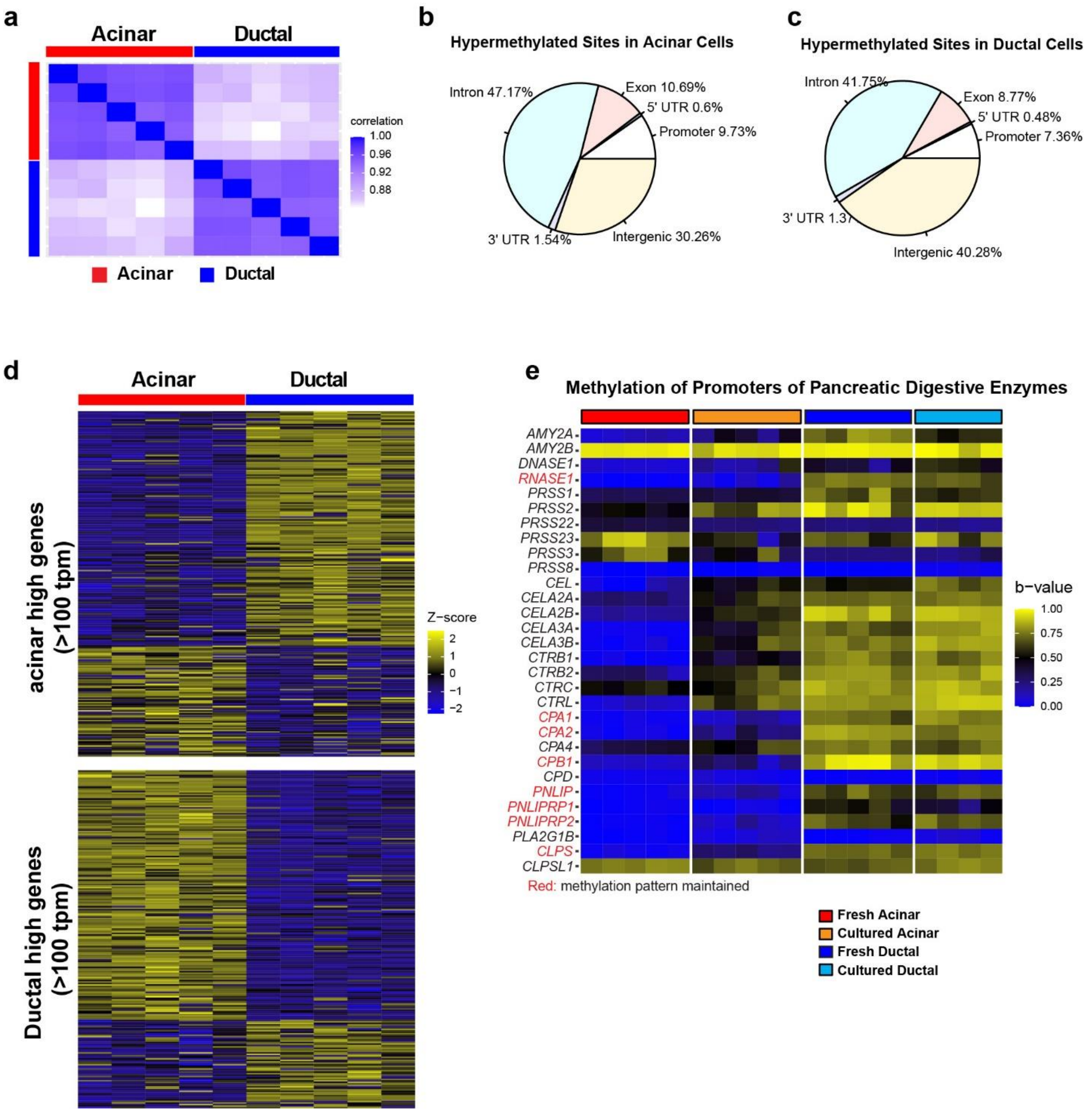

Figure S2. DNA methylation status of fresh and cultured acinar and ductal cells.

a. Correlation of DNA methylation of fresh acinar (n=5) and ductal cells (n=5) by all surveyed methylation sites. **b-c.** Distribution of lineage-specific differentially methylated CpG sites with reference to genomic features. **d.** Heatmap of promoter methylation status of top differentially expressed genes (identified in Figure 1) in fresh acinar and ductal samples. **e.** Heatmap of DNA methylation status in the promoter regions of genes

encoding pancreatic digestive enzymes of fresh acinar (n=5), cultured acinar (n=5), fresh ductal (n=5) and cultured ductal cells (n=4). Genes in red are associated with a lineage-specific methylation pattern which is maintained after long term culture.

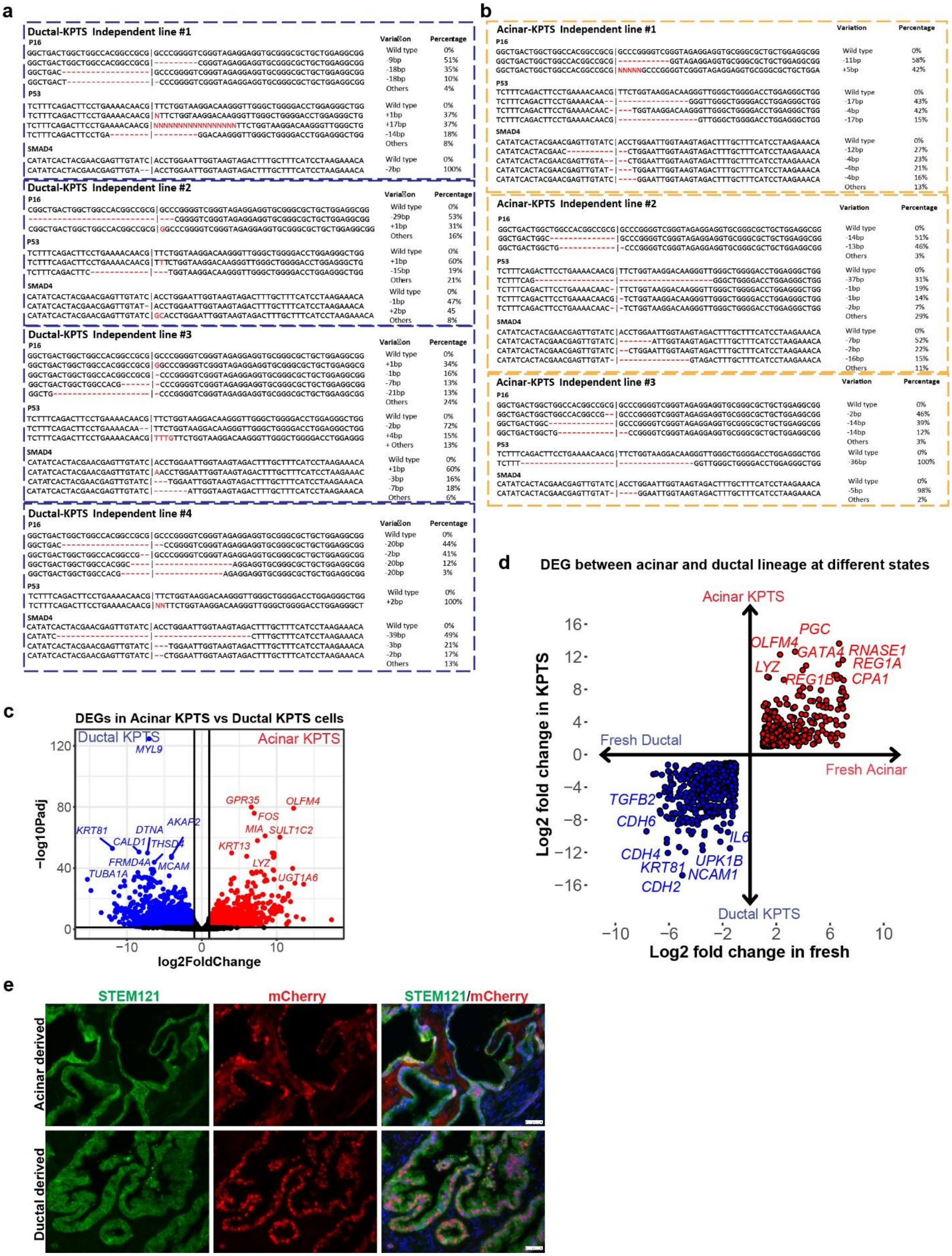
a
Ductal-KPTS Independent line #1
Ductal-KPTS Independent line #2
Ductal-KPTS Independent line #3
Ductal-KPTS Independent line #4
P16
P53
SMAD4
Percentage
Wild type
Others
b
Acinar-KPTS Independent line #1
Acinar-KPTS Independent line #2
Acinar-KPTS Independent line #3
Variation
Percentage
c
DEGs in Acinar KPTS vs Ductal KPTS cells
Ductal KPTS
Acinar KPTS
−log10Padj
log2FoldChange
MYL9
KRT81
CALD1
DTNA
AKAP2
THSD4
FRMD4A
MCAM
TUBA1A
GPR35
OLFM4
FOS
MIA
SULT1C2
KRT13
LYZ
UGT1A6
d
DEG between acinar and ductal lineage at different states
Acinar KPTS
Ductal KPTS
Fresh Ductal
Fresh Acinar
Log2 fold change in KPTS
Log2 fold change in fresh
PGC
OLFM4
GATA4
RNASE1
REG1A
LYZ
REG1B
CPA1
TGFB2
CDH6
IL6
CDH4
UPK1B
KRT81
NCAM1
CDH2
e
STEM121
mCherry
STEM121/mCherry
Acinar derived
Ductal derived

Figure S3. Genetical engineering primary acinar and ductal cells to oncogenic KPTS cells. a-b. Genotyping of *p16*, *p53* and *SMAD4* mutation status of four ductal-KPTS 2D cultures (a) and three acianr-3D cultures (b). **c.** Volcano plot of the differentially expressed genes in acinar-KPTS (n=3) versus ductal-KPTS cells (n=4, fold change > 2, adj.p < 0.05, by negative binomial Wald test using DESeq2 package in R software). **d.** Scatter plot of genes which were significantly highly expressed in both fresh and KPTS cells of each lineage compared with the other (log2 fold change > 1, adj.p < 0.05). Acinar high genes are colored in red, ductal high genes are colored in blue. X axis represents log2 fold change between fresh acinar and fresh ductal, y axis represents log2 fold change between acinar-KPTS and ductal-KPTS. **e.** Immunofluorescence staining of human specific STEM121 and mCherry of acinar-derived and ductal-derived tumor sections. The staining was performed in 6 independent tumor tissues with 3 sections for each. Scale bar: 50 µm.

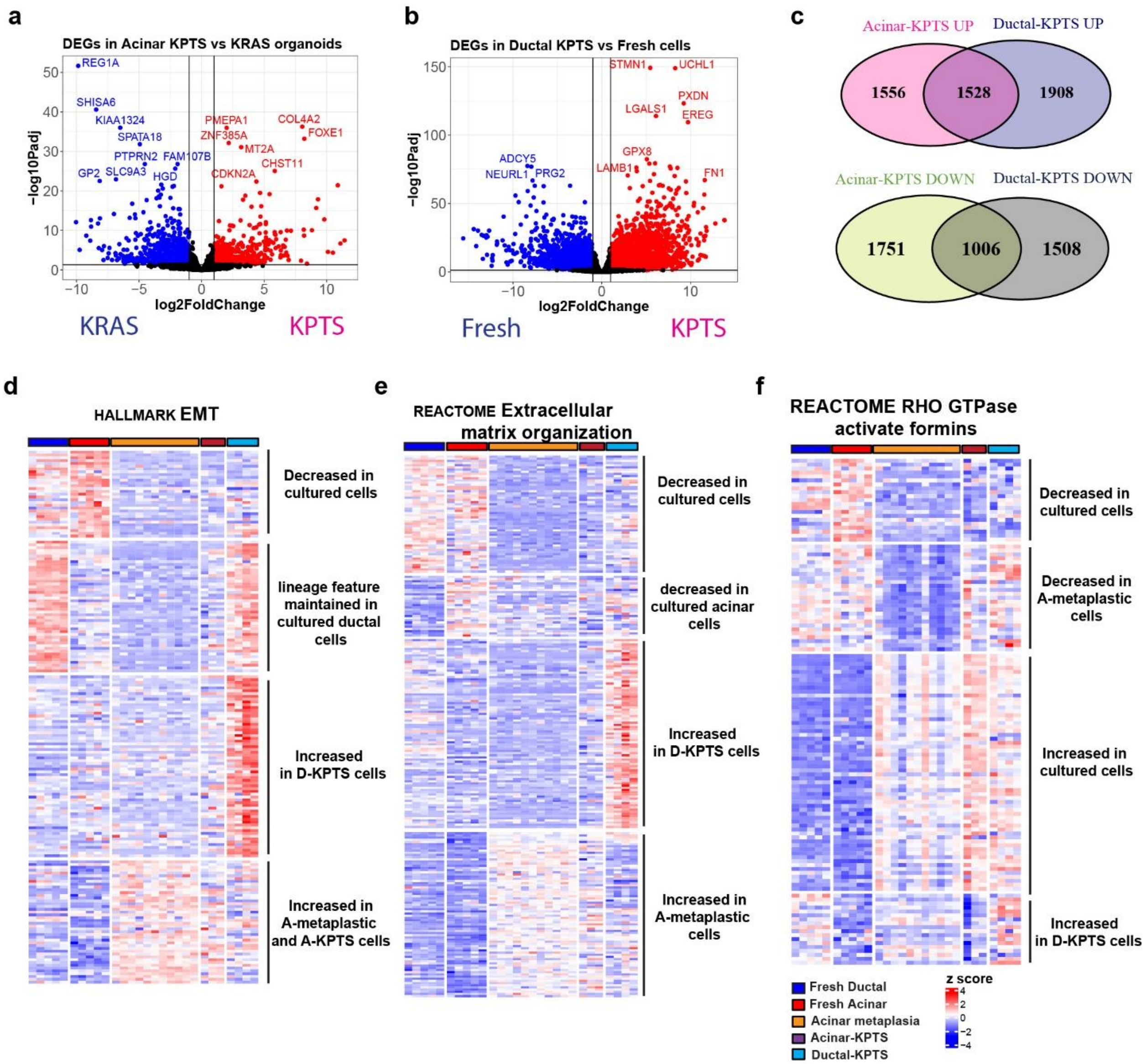

Figure S4. Molecular profiling oncogenic KPTS cells of acinar and ductal lineages. a. Volcano plot of the differentially expressed genes in acinar-KPTS (n=3) versus acinar-KRAS organoids (n=5). DEGs were identified by negative binomial Wald test using DESeq2 1.36.0 in R software with fold change > 2 and adjusted p-value < 0.05. **b.** Volcano plot of the DEGs in ductal-KPTS (n=4) versus fresh ductal cells (n=5). DEGs were identified by negative binomial Wald test using DESeq2 with fold change > 2 and adjusted p-value < 0.05. **c.** Intersection of DEGs (described in a and b) in acinar-

KPTS and ductal-KPTS cells. **d-f.** Expression heatmaps of genes in indicated gene sets in fresh, metaplastic, and KPTS cells. K means clustering was performed to identify different expression patterns across all groups of samples.

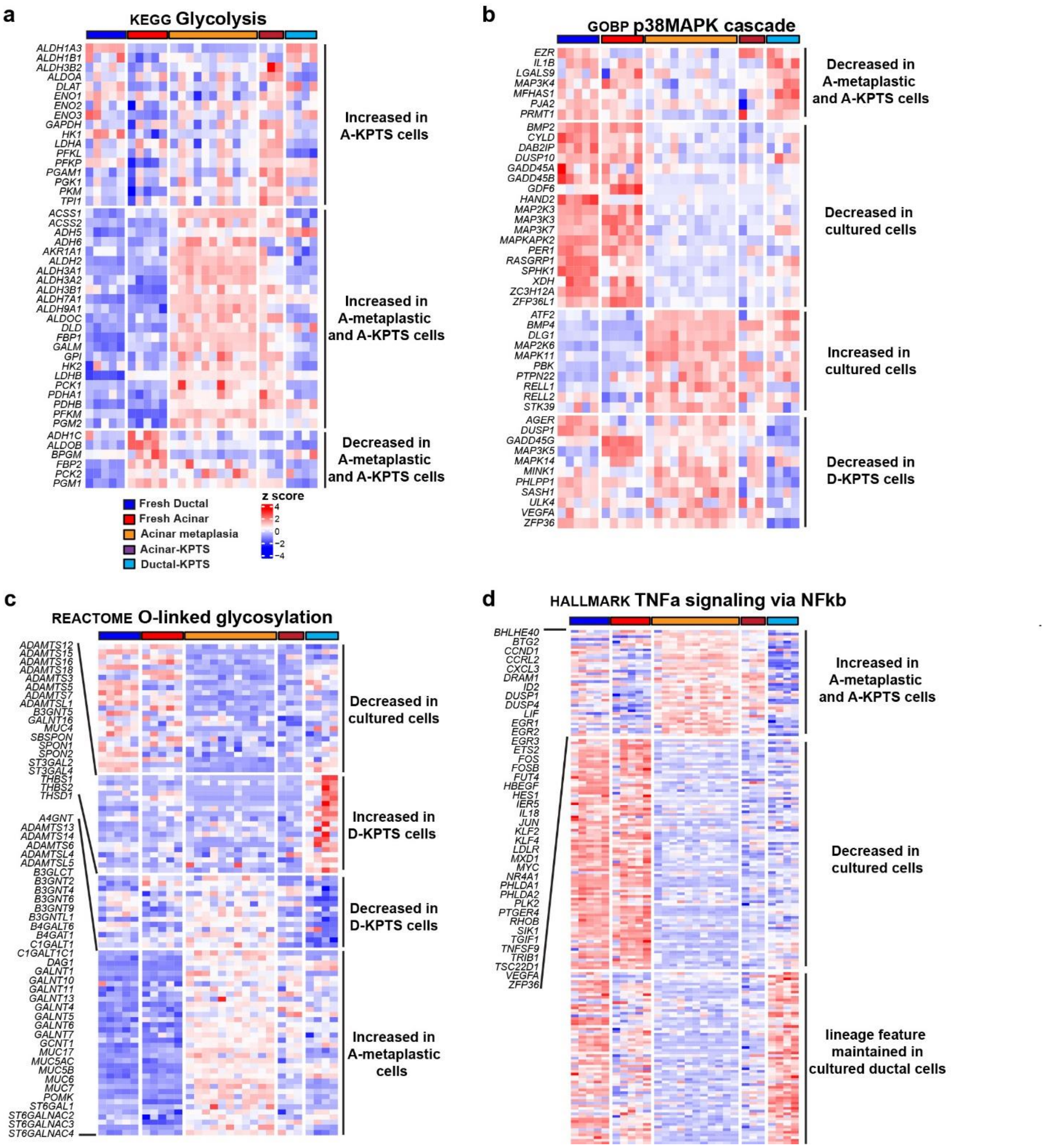

Figure S5. Altered transcriptomic programs during oncogenic transition. a-d. Expression heatmaps of genes in indicated gene sets in fresh, metaplastic, and KPTS cells. Fresh ductal n = 5, fresh acinar n = 5, acinar-metaplastic n = 11, acinar-KPTS n =

3, ductal-KPTS n = 4. K means clustering was performed to identify different expression patterns across all groups of samples.

a

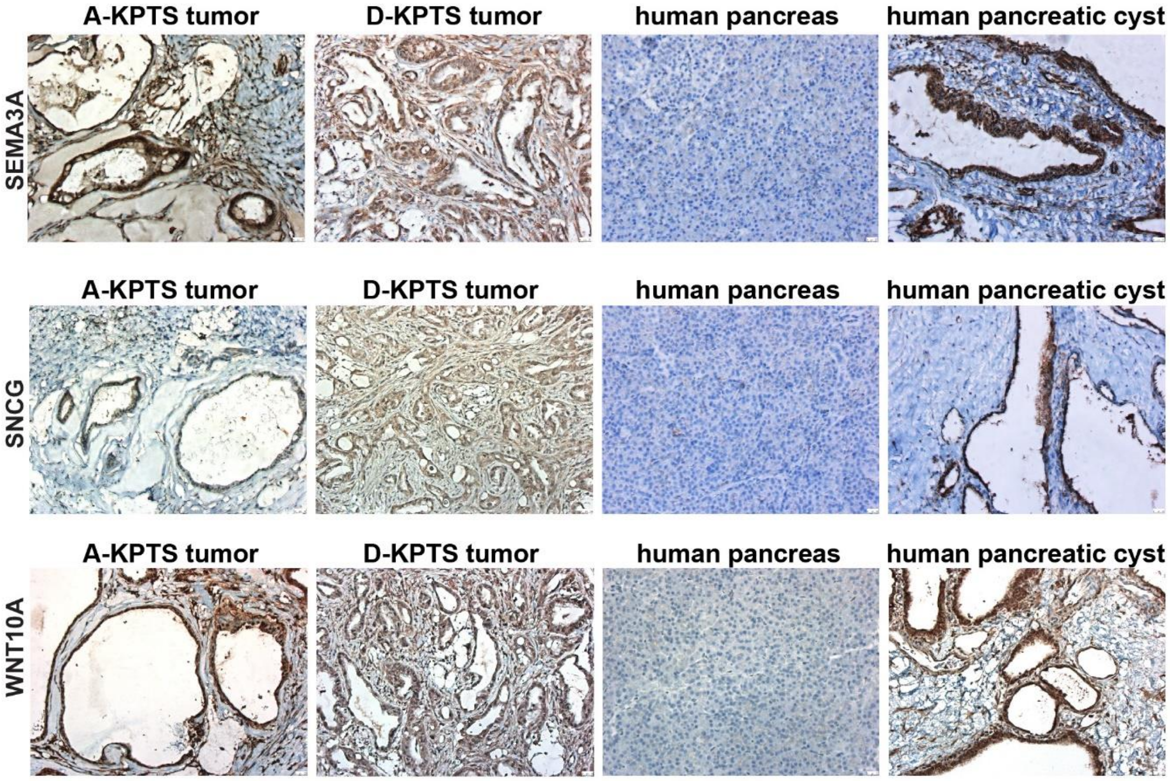

b

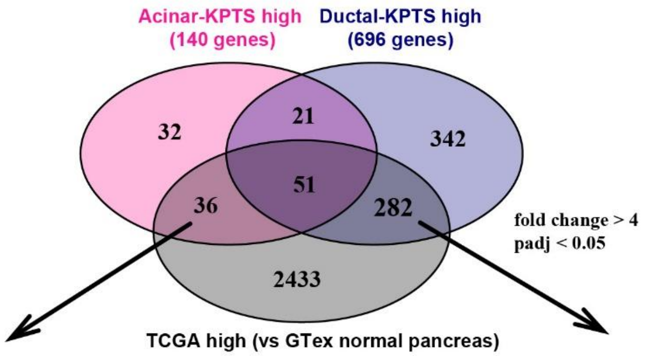

Acinar-KPTS high & TCGA high (36 genes)

	Extracellular space and Plasma membrane	Transcription Factor and others
**Associated with PDAC known function**	*CSTB, IL10, NTSR1, *PSCA, S100A4,*	*CDC25C,SIX1, *UBE2C*
**Associated with PDAC unclear function**	*ITGB2, LYPD3, SLC16A3*	*ACSL5, SDR16C5, TBX15*
**Unknown implication in PDAC**	*CD22, CILP2, FAM3D, FIBCD1, LGR6, NPFFR1*	*ACTL8, ALDH3B2, FNDC11, DMBX1, FABP6, GREB1L, GSDMA, HPGD, KRT79, PTTG1, S100A5, ST8SIA6, TPRG1*

Ductal-KPTS high & TCGA high (38 out of 282 genes)

	Extracellular space	Transcription Factor
**Associated with PDAC known function**	*BDNF, DKK2, FGF1, FGF19, FGF5, GREM1, INHBA, LGALS1, LTBP1, MDK, *NOG, NRP2, PTHLH, SERPINB2, SERPINE1, TGFB2, THBS1, THBS2, VEGFC, WNT7A,WNT7B*	*E2F7, GATA3, ISL2, RUNX2, HMGA2, HOXB8, HOXB9, *LOXL2*
**Associated with PDAC unclear function**	*BMP3, CFH, LTBP2*	*SIM2, BASP1,*
**Unknown implication in PDAC**	*GDF5*	*HES2, PRRX2, ZFHX4*

*** : Prognostic genes from TCGA PAAD**

Figure S6. Identified genes upregulated in early and established PDAC. a. Immunohistochemistry staining of SEMA3A, SNCG and WNT10A proteins in the KPTS tumors generated in this work (n = 2 with 8 field of views for each), and a human pancreatic cystic neoplasm sample as well as adjacent normal pancreas (8 field of views for each). Scale bar: 25 µm. **b.** Annotation of 36 genes highly expressed in both acinar-KPTS cells and TCGA PDAC samples (left), as well as selected genes highly expressed in both ductal-KPTS cells and TCGA PDAC samples (right).
