# Supplementary material for: Reconstitution of human PDAC using primary cells reveals oncogenic transcriptomic features at tumor onset
Source: Nat Commun. 2024 Jan 27;15:818. doi: 10.1038/s41467-024-45097-2 (PMC10821902; doi:10.1038/s41467-024-45097-2)
Supplement: Supplementary file 3 — Description of Additional Supplementary Files [file 41467_2024_45097_MOESM3_ESM.pdf]

Description of Additional Supplementary Files

Supplementary Data 1. Organ donor information of the primary tissues used in this study.

Supplementary Data 2. Six subgroups of DEGs described in Figure 1d and their expression (normalized and transformed using DESeq2 1.36.0).

Supplementary Data 3. List of genes described in Figure 3d and their log2 normalized counts.

Supplementary Data 4. List of genes described in Figure S3d and their log2 fold change.

Supplementary Data 5. Result tables of differential expression analysis from acinar-KPTS vs acinar-KRAS cells (Figure S4a), and ductal-KPTS vs fresh ductal (Figure S4b), respectively. DEGs were identified by negative binomial Wald test using DESeq2 1.36.0 in R software with fold change > 2 and adjusted p-value < 0.05.

Supplementary Data 6. List of genes described in Figure 6a-c, including 140 genes significantly highly expressed in acinar-KPTS cells (compared with fresh and metaplastic cells), 696 genes significantly highly expressed in ductal-KPTS cells (compared with fresh and metaplastic cells), and 2805 genes significantly highly expressed in TCGA PDAC samples (compared with GTEx normal pancreas).
