# Supplementary material for: Reconstitution of human PDAC using primary cells reveals oncogenic transcriptomic features at tumor onset
Source: Nat Commun. 2024 Jan 27;15:818. doi: 10.1038/s41467-024-45097-2 (PMC10821902; doi:10.1038/s41467-024-45097-2)
Supplement: Supplementary file 10 — Reporting Summary [file 41467_2024_45097_MOESM10_ESM.pdf]

nature portfolio

Corresponding author(s): Jun Liu, Pei Wang

Last updated by author(s): Yi Xu, Pei Wang November 30, 2023

Reporting Summary

Nature Portfolio wishes to improve the reproducibility of the work that we publish. This form provides structure for consistency and transparency in reporting. For further information on Nature Portfolio policies, see our Editorial Policies and the Editorial Policy Checklist.

Please do not complete any field with "not applicable" or n/a. Refer to the help text for what text to use if an item is not relevant to your study.
For final submission: please carefully check your responses for accuracy; you will not be able to make changes later.

Statistics

For all statistical analyses, confirm that the following items are present in the figure legend, table legend, main text, or Methods section.

n/a	Confirmed	
[ ]	[X]	The exact sample size (n) for each experimental group/condition, given as a discrete number and unit of measurement
[ ]	[X]	A statement on whether measurements were taken from distinct samples or whether the same sample was measured repeatedly
[ ]	[X]	The statistical test(s) used AND whether they are one- or two-sided<br>*Only common tests should be described solely by name; describe more complex techniques in the Methods section.*
[X]	[ ]	A description of all covariates tested
[ ]	[X]	A description of any assumptions or corrections, such as tests of normality and adjustment for multiple comparisons
[ ]	[X]	A full description of the statistical parameters including central tendency (e.g. means) or other basic estimates (e.g. regression coefficient) AND variation (e.g. standard deviation) or associated estimates of uncertainty (e.g. confidence intervals)
[ ]	[X]	For null hypothesis testing, the test statistic (e.g. F, t, r) with confidence intervals, effect sizes, degrees of freedom and P value noted<br>*Give P values as exact values whenever suitable.*
[X]	[ ]	For Bayesian analysis, information on the choice of priors and Markov chain Monte Carlo settings
[X]	[ ]	For hierarchical and complex designs, identification of the appropriate level for tests and full reporting of outcomes
[ ]	[X]	Estimates of effect sizes (e.g. Cohen's d, Pearson's r), indicating how they were calculated

Our web collection on statistics for biologists contains articles on many of the points above.

Software and code

Policy information about availability of computer code

Data collection	Flow sorting was performed using a FACSAriaTM II, Electron Microscopy images were captured using a JEOL 1230 electron microscope, flow cytometry data was collected using a BD LSRII, IF and IHC images were captured using a Leica DMI6000 B microscope
Data analysis	TopHat 2.1.1 , HTSeq 0.11.1, DESeq2 1.36.0 , clusterProfiler 4.4.4 , limma 3.52.4, Seurat 4.3.0.1, MuSiC 1.0.0, Bismark 0.19.1, FlowJo v10

For manuscripts utilizing custom algorithms or software that are central to the research but not yet described in published literature, software must be made available to editors and reviewers. We strongly encourage code deposition in a community repository (e.g. GitHub). See the Nature Portfolio guidelines for submitting code & software for further information.

Data

Policy information about availability of data

All manuscripts must include a data availability statement. This statement should provide the following information, where applicable:

- Accession codes, unique identifiers, or web links for publicly available datasets
- A description of any restrictions on data availability
- For clinical datasets or third party data, please ensure that the statement adheres to our policy

Data Availability

The raw and processed high throughput sequencing data generated in this study have been deposited and publicly available at Gene Expression Omnibus under accession numbers GSE223153 [https://www.ncbi.nlm.nih.gov/geo/query/acc.cgi?acc=GSE223153] and GSE222990 [https://www.ncbi.nlm.nih.gov/geo/query/acc.cgi?acc=GSE222990]. Raw read counts and metadata of referenced scRNA-seq datasets used in this study are available for download according to the authors' instructions from corresponding references15,19,20,24, including GSE172380 [https://0-www-ncbi-nlm-nih-gov.brum.beds.ac.uk/geo/query/acc.cgi?acc=GSE172380], GSE141017 [https://www.ncbi.nlm.nih.gov/geo/query/acc.cgi?acc=GSE141017], [http://singlecell.charite.de/cellbrowser/pancreas/], and HTAN Data Coordinating Center Data Portal under the HTAN WUSTL Atlas [https://data.humantumoratlas.org/], respectively. GTEx normal pancreas RNA expression data can be accessed from GTEx Portal [https://gtexportal.org/home/downloads/adult-gtex#bulk_tissue_expression]. TCGA pancreatic cancer RNA expression data are available at GDC data portal [https://portal.gdc.cancer.gov/projects/TCGA-PAAD]. Human reference genome GRCh38 is available at [https://hgdownload.soe.ucsc.edu/goldenPath/hg38/bigZips/].

Research involving human participants, their data, or biological material

Policy information about studies with human participants or human data. See also policy information about sex, gender (identity/presentation), and sexual orientation and race, ethnicity and racism.

Reporting on sex and gender	Gender of human donors was provided in supplementary data 1, and was not considered in the study design
Reporting on race, ethnicity, or other socially relevant groupings	Ethnicity of human donors was provided in supplementary data 1, and was not considered in the study design
Population characteristics	Human primary pancreatic tissues were obtained from 21 organ donors deceased due to acute traumatic or anoxic death, with the ages range from 22 to 56. Human pancreatic cystic neoplasm sample was collected from a 71-year old black male
Recruitment	No selection bias present in this study to impact results.
Ethics oversight	All experiments involving normal human primary pancreatic tissues from organ donors were reviewed by the institutional review board at UT Health San Antonio. The tissues were de-identified, with only information on sex, race, age, weight, height and cause of death. The IRB committee has agreed that the project does not require IRB approval. Human pancreatic cystic neoplasm sample was collected and prepared at UT Health at San Antonio with patient's consent in accordance with the guidelines. Experiment involving this clinical human PDAC sample was approved by the institutional review board at UT Health San Antonio (IRB protocol #20230412NHR).

Note that full information on the approval of the study protocol must also be provided in the manuscript.

Field-specific reporting

Please select the one below that is the best fit for your research. If you are not sure, read the appropriate sections before making your selection.

[X] Life sciences [ ] Behavioural & social sciences [ ] Ecological, evolutionary & environmental sciences

For a reference copy of the document with all sections, see nature.com/documents/nr-reporting-summary-flat.pdf

Life sciences study design

All studies must disclose on these points even when the disclosure is negative.

Sample size	Sample size was not calculated before experiment. The sample size was determined based on the availability of human primary samples to provide at least 3 biological replicate for a sufficient statistical power.
Data exclusions	No data exclusion involved in this work
Replication	3 to 6 biological replicates of experiments using human primary tissues were performed. All attempts at replication were successful
Randomization	When comparing samples from different groups, we used paired samples from the same donor tissues whenever possible. When paired sample is not available, we make sure to include multiple biological replicates to minimize potential heterogeneity.
Blinding	All the human tissues were shipped to us with donors already de-identified. All the experimental process and following data analysis were performed by following universal protocols or objective quantitative methods, so we were not blinded to sample allocation.

Behavioural & social sciences study design

All studies must disclose on these points even when the disclosure is negative.

Study description	
Research sample	
Sampling strategy	
Data collection	
Timing	
Data exclusions	
Non-participation	
Randomization	

Ecological, evolutionary & environmental sciences study design

All studies must disclose on these points even when the disclosure is negative.

Study description

Research sample

Sampling strategy

Data collection

Timing and spatial scale

Data exclusions

Reproducibility

Randomization

Blinding

Did the study involve field work? ☐ Yes ☐ No

Field work, collection and transport

Field conditions

Location

Access & import/export

Disturbance

Reporting for specific materials, systems and methods

We require information from authors about some types of materials, experimental systems and methods used in many studies. Here, indicate whether each material, system or method listed is relevant to your study. If you are not sure if a list item applies to your research, read the appropriate section before selecting a response.

Materials & experimental systems

n/a	Involved in the study	
☐	X	Antibodies
X	☐	Eukaryotic cell lines
X	☐	Palaeontology and archaeology
☐	X	Animals and other organisms
X	☐	Clinical data
X	☐	Dual use research of concern
X	☐	Plants

Methods

n/a	Involved in the study	
X	☐	ChIP-seq
☐	X	Flow cytometry
X	☐	MRI-based neuroimaging

Antibodies

Antibodies used

Validation

Antibodies used for immuno-staining:
Mouse anti human STEM121 (Takara Bio, #Y40410), dilution 1:50;
Mouse anti human/mouse/rat KRT19 (DSHB, #Troma-III), dilution 1:50;
Rabbit anti human Alpha-Amylase (Sigma Aldrich, #A8273), dilution 1:500;
Mouse anti human Mucin5AC (Santa Cruz Biotechnology, #sc-33667), dilution 1:50;
Rabbit anti human/mouse WNT10A (Thermo Fisher, #26238-1-AP), dilution 1:50;
Rabbit anti human AHNAK2 (Sigma Aldrich, #HPA002940), dilution 1:200;
Mouse anti human/mouse/rat AREG (Santa Cruz Biotechnology, #SC-74501), dilution 1:50;
Rabbit anti human/mouse/rat SEMA3A (Abcam, #AB199475), dilution 1:300;
Mouse anti human/mouse/rat SNCG (Santa Cruz Biotechnology, #SC-65979), dilution 1:50;
Alexa Fluor® 488-conjugated AffiniPure Donkey Anti-Mouse IgG (H+L) (Jackson ImmunoResearch, #715-545-150), dilution 1:250;
Cy™3-conjugated AffiniPure Donkey Anti-Rat IgG (H+L) (Jackson ImmunoResearch, #712-165-150), dilution 1:250;
Alexa Fluor® 647-conjugated AffiniPure Donkey Anti-Rabbit IgG (Jackson ImmunoResearch, #711-605-152), dilution 1:250;
Biotinylated-Goat Anti-Mouse Ig (Multiple Adsorption) (BD Biosciences, #550337), dilution 1:100.
Antibodies used for flow cytometry: FITC-conjugated UEA-1 (Vector Laboratories, FL-1061-5), Pacific blue-conjugated anti-CLA (BioLegend, 321308), FITC anti-human HLA-A,B,C antibody (BioLegend, 311403), FITC anti-human HLA-DR, DP, DQ antibody (BioLegend, 361705), FITC Mouse IgG2a isotype control (BioLegend, 400209)
Validation:
All the antibodies used in this study are commercially available and have been validated by their respective manufacturers, with the detailed validation data available on the corresponding websites.

Eukaryotic cell lines

Policy information about cell lines and Sex and Gender in Research

Cell line source(s)	
Authentication	
Mycoplasma contamination	
Commonly misidentified lines (See ICLAC register)	

Palaeontology and Archaeology

Specimen provenance	
Specimen deposition	
Dating methods	

☐ Tick this box to confirm that the raw and calibrated dates are available in the paper or in Supplementary Information.

Ethics oversight	

Note that full information on the approval of the study protocol must also be provided in the manuscript.

Animals and other research organisms

Policy information about studies involving animals; ARRIVE guidelines recommended for reporting animal research, and Sex and Gender in Research

Laboratory animals	Four to six-week old NOD scid gamma mice (Strain #:005557) were purchased from The Jackson Laboratory. The mice were housed five per cage in a pathogen-free system with water and food ad libitum, with 12 hours day/night cycle at 20-25 °C and 50-60% humidity
Wild animals	No wild animals are used in this study
Reporting on sex	The animal sex was reported in the Methods section, and was not considered in the study design and analysis, as no evidence for the impact of sex on the subcutaneous tumorigenesis of pancreatic cancer cells.
Field-collected samples	No field collected samples are used in this study
Ethics oversight	All animal experiments were approved by the Institutional Animal Care and Use Committees at UT Health San Antonio (protocol #20130023AR), and were performed in accordance with relevant guidelines and regulations.

Note that full information on the approval of the study protocol must also be provided in the manuscript.

Clinical data

Policy information about clinical studies

All manuscripts should comply with the ICMJE guidelines for publication of clinical research and a completed CONSORT checklist must be included with all submissions.

Clinical trial registration	
Study protocol	
Data collection	
Outcomes	

Dual use research of concern

Policy information about dual use research of concern

Hazards

Could the accidental, deliberate or reckless misuse of agents or technologies generated in the work, or the application of information presented in the manuscript, pose a threat to:

No | Yes

- [ ] [ ] Public health
- [ ] [ ] National security
- [ ] [ ] Crops and/or livestock
- [ ] [ ] Ecosystems
- [ ] [ ] Any other significant area

Experiments of concern

Does the work involve any of these experiments of concern:

No | Yes

- [ ] [ ] Demonstrate how to render a vaccine ineffective
- [ ] [ ] Confer resistance to therapeutically useful antibiotics or antiviral agents
- [ ] [ ] Enhance the virulence of a pathogen or render a nonpathogen virulent
- [ ] [ ] Increase transmissibility of a pathogen
- [ ] [ ] Alter the host range of a pathogen
- [ ] [ ] Enable evasion of diagnostic/detection modalities
- [ ] [ ] Enable the weaponization of a biological agent or toxin
- [ ] [ ] Any other potentially harmful combination of experiments and agents

Plants

Seed stocks

Novel plant genotypes

Authentication

ChIP-seq

Data deposition

- [ ] Confirm that both raw and final processed data have been deposited in a public database such as GEO.
- [ ] Confirm that you have deposited or provided access to graph files (e.g. BED files) for the called peaks.

Data access links
May remain private before publication.

Files in database submission

Genome browser session
(e.g. UCSC)

Methodology

Replicates

Sequencing depth

Antibodies

Peak calling parameters

Data quality

Software

Flow Cytometry

Plots

Confirm that:

- [X] The axis labels state the marker and fluorochrome used (e.g. CD4-FITC).
- [X] The axis scales are clearly visible. Include numbers along axes only for bottom left plot of group (a 'group' is an analysis of identical markers).
- [X] All plots are contour plots with outliers or pseudocolor plots.
- [ ] A numerical value for number of cells or percentage (with statistics) is provided.

Methodology

Sample preparation	Sample preparation for flow cytometry analysis and sorting are described in details in the Method section
Instrument	FACS AriaTM II (BD Biosciences) for flow sorting, BD LSRII (BD Biosciences) for flow analysis
Software	Flowjo v10
Cell population abundance	
Gating strategy	

- [ ] Tick this box to confirm that a figure exemplifying the gating strategy is provided in the Supplementary Information.

Magnetic resonance imaging

Experimental design

Design type	
Design specifications	
Behavioral performance measures	

Imaging type(s)	
Field strength	
Sequence & imaging parameters	
Area of acquisition	

Diffusion MRI [ ] Used [ ] Not used

Preprocessing

Preprocessing software	
Normalization	
Normalization template	
Noise and artifact removal	
Volume censoring	

Statistical modeling & inference

Model type and settings	
Effect(s) tested	

Specify type of analysis: [ ] Whole brain [ ] ROI-based [ ] Both

Statistic type for inference

(See Eklund et al. 2016)

Correction

Models & analysis

n/a	Involved in the study
☐	☐ Functional and/or effective connectivity
☐	☐ Graph analysis
☐	☐ Multivariate modeling or predictive analysis

Functional and/or effective connectivity

Graph analysis

Multivariate modeling and predictive analysis

This checklist template is licensed under a Creative Commons Attribution 4.0 International License, which permits use, sharing, adaptation, distribution and reproduction in any medium or format, as long as you give appropriate credit to the original author(s) and the source, provide a link to the Creative Commons license, and indicate if changes were made. The images or other third party material in this article are included in the article's Creative Commons license, unless indicated otherwise in a credit line to the material. If material is not included in the article's Creative Commons license and your intended use is not permitted by statutory regulation or exceeds the permitted use, you will need to obtain permission directly from the copyright holder. To view a copy of this license, visit http://creativecommons.org/licenses/by/4.0/
